# Plasmodium SEY1 is a novel druggable target that contributes to imidazolopiperazine mechanism of action

**DOI:** 10.21203/rs.3.rs-4892449/v1

**Published:** 2024-09-23

**Authors:** Elizabeth Winzeler, Krypton Carolino, Mariana Laureano De Souza, Daisy Chen, Jean-Claude Farre, James Blauwkamp, Sabrina Absalon, Sonja Ghidelli-Disse, Alexander Morano, Jeffrey Dvorin, Maria Jose Lafuente-Monasterio, Francisco-Javier Gamo

**Affiliations:** University of California, San Diego; University of California, San Diego; University of California, San Diego; University of California, San Diego; University of California, San Diego; Boston Children’s Hospital and Harvard Medical School

## Abstract

The precise mode of action of ganaplacide (KAF156), a phase III antimalarial candidate, remains elusive. Here we employ omics-based methods with the closely related chemical analog, GNF179, to search for potential *Plasmodium* targets. Ranking potential targets derived from chemical genetics and proteomic affinity chromatography methodologies identifies *SEY1*, or Synthetic Enhancement of YOP1, which is predicted to encode an essential dynamin-like GTPase implicated in homotypic fusion of endoplasmic reticulum (ER) membranes. We demonstrate that GNF179 decreases *Plasmodium* SEY1 melting temperature. We further show that GNF179 binds to recombinant *Plasmodium* SEY1 and subsequently inhibits its GTPase activity, which is required for maintaining ER architecture. Using ultrastructure expansion microscopy, we find GNF179 treatment changes parasite ER and Golgi morphology. We also confirm that *SEY1* is an essential gene in *P. falciparum*. These data suggest that *SEY1* may contribute to the mechanism of action of imidazolopiperazines and is a new and attractive druggable target.

## One sentence summary

The antimalarial effects of ganaplacide analog GNF179 are associated with the inhibition of the *Plasmodium dynamin*-like SEY1 GTPase activity, which is important for shaping the parasite endoplasmic reticulum.

## Introduction

Malaria, caused by the *Plasmodium* genus of organisms, remains the most common infectious parasitic disease worldwide. In 2022, there were 249 million cases, an annual increase of two million; of these cases, 608,000 were fatal (WHO, 2023). Despite public health efforts to monitor malaria transmission (Pattanshetty et al., 2024) and increase access to prevention and treatment (Mao et al., 2023), the disease remains a significant burden due to ineffective vaccines (Duffy, 2022) and the ever-increasing insecticide and antimalarial resistance, including the gold standard artemisinin-based combination therapies (Siqueira-Neto et al., 2023). Resistance has been reported against nearly all antimalarial classes – endoperoxides (Dondorp et al., 2009), quinolines (Sidhu et al., 2002), antifolates, naphthoquinones (Antony & Parija, 2016). Thus, there is a dire need for medicines possessing novel modes of action (MOA) to combat the antimalarial resistance arms race. If malaria were ever to be eradicated, antimalarials that inhibit growth at all stages of the complex parasite life cycle is ideal (Luo et al., 2023).

Imidazolopiperazines (IZPs), discovered through target-agnostic phenotypic screens conducted by the Genomics Institute of the Novartis Research Foundation in 2007 (Meister et al., 2011), are a new generation of antimalarial compounds that possess a novel mode of action. The potency against *Plasmodium* and the physiochemical and pharmacokinetic properties of the initial screening hit was improved through medicinal chemistry efforts, leading to the development of ganaplacide, or KAF156 (Wu et al., 2011). This class is the first next-generation compound series to be highly active against multiple stages of the parasite life cycle – asexual blood stage, liver stage (Meister et al., 2011), sexual blood stage, and parasite transmission (Kuhen et al., 2014; Ouologuem et al., 2022) – while maintaining low toxicity against human host cells. The series is even active against artemisinin-resistant parasites (Dembele et al., 2018; Yipsirimetee et al., 2022). Human clinical trials support its properties of quick absorption, long half-life, and minimal adverse effects (Leong et al., 2014), and its ability to clear acute *P. falciparum* and *P. vivax* malaria infections (White et al., 2016). IZPs also display prophylactic activity in controlled human malaria infection studies (Kublin et al., 2021). KAF156 has been shown to work effectively in combination with piperaquine (Leong et al., 2018) and lumefantrine (Ogutu et al., 2023), and it is currently in the phase III patient exploration stage alongside the latter (Siqueira-Neto et al., 2023).

Despite the detailed clinical studies, IZP mode of action remains poorly characterized. Profiling of 113 metabolites in *P. falciparum* treated with KAF156 did not present clear metabolomic perturbations (Allman et al., 2016). *In-vitro* evolution and whole genome analysis (IVIEWGA) of individual *P. falciparum* clones evolved to confer resistance to closely related chemical analog GNF179 has consistently yielded single nucleotide variants (SNVs) in three genes: *PfCARL* (PF3D7_0321900), cyclic amine resistance transporter (LaMonte et al., 2016); *PfUGT* (PF3D7_1113300), UDP-galactose transporter; *PfACT* (PF3D7_1036800), acetyl-CoA transporter (Lim et al., 2016). *PfCARL* and *PfUGT* are predicted to be essential in *Plasmodium* through a lack of gene disruptions by *piggyBac* transposon insertions (Zhang et al., 2018). Although mutations in *PfUGT* are rare, 13 unique mutations in *PfCARL* have been identified, many of which were further validated via CRISPR/Cas9 genetic engineering (LaMonte et al., 2016). *PfCARL* yeast ortholog, Saccharomyces cerevisiae *EMP65*, protects folding polypeptides from promiscuous degradation within the endoplasmic reticulum (ER) lumen (Zhang et al., 2017), while human ortholog *HsTAPT* is implicated in Golgi morphology and trafficking (Symoens et al., 2015). *PfUGT* encodes a solute carrier 35 (SLC35) member that assists in sugar import to the ER (Ishida & Kawakita, 2004). *PfACT* imports acetyl-CoA to the ER for use in lysine acetylation of some newly synthesized proteins (Nyonda et al., 2022). Unfortunately, mutations in these genes also confer resistance to unrelated compound classes, suggesting that they encode multidrug resistance transporters rather than IZP targets (Imlay et al., 2023; Lim et al., 2016; Magistrado et al., 2016). These results predict IZPs to inhibit protein trafficking, protein transport, and establishment of new permeation pathways (LaMonte et al., 2020).

Further IVIEWGA experimentation with GNF179 on the *S. cerevisiae* model organism yielded adaptive mutations in genes also functioning within the ER, specifically implicated in ER-based lipid homeostasis and autophagy: *ScELO2* contributes to sphingolipid biosynthesis; *ScSUR2* and *ScLCB4* are involved in sphingolipid metabolism; *ScATG15* is induced in proteasome-independent ER expansion caused by misfolded protein aggregation; *ScSEC66* is part of the *ScSEC63* complex involved in protein targeting and import into the ER. However, *ScSUR2* and *ScELO2* also appear to be multidrug resistance genes in yeast, and some of these gene orthologs are nonessential in *Plasmodium* (LaMonte et al., 2020). Thus, the search continues.

Here we identify and validate *SEY1* as a possible biological target of IZPs. SEY1, or Synthetic Enhancer of YOP1, is predicted to be an essential dynamin-like GTPase implicated in homotypic fusion of ER membranes. It is structurally conserved, including within the druggable GTPase domain. We detect elevated levels of *PfSEY*1 in pulldown experiments with GNF179 and elevated levels of GNF179 on *PvSEY*1-coated sensor chips in surface plasmon resonance experiments. GNF179 also reduces *PvSEY*1 melting temperature, adding further evidence of interaction. GNF179 inhibits *PvSEY*1 GTPase activity, supporting molecular docking predictions of binding to conserved GTPase motifs. These results may explain our observed parasite ER morphology changes upon GNF179 treatment. We also demonstrate that *Plasmodium SEY1* overexpression confers resistance to GNF179, while *Plasmodium SEY1* knockdown confers sensitivity to the compound. Finally, we confirm that *PfSEY1* is an essential parasite gene.

## Results

### Pf SEY1 is pulled down abundantly by GNF179-linked beads

**Figure 1.**
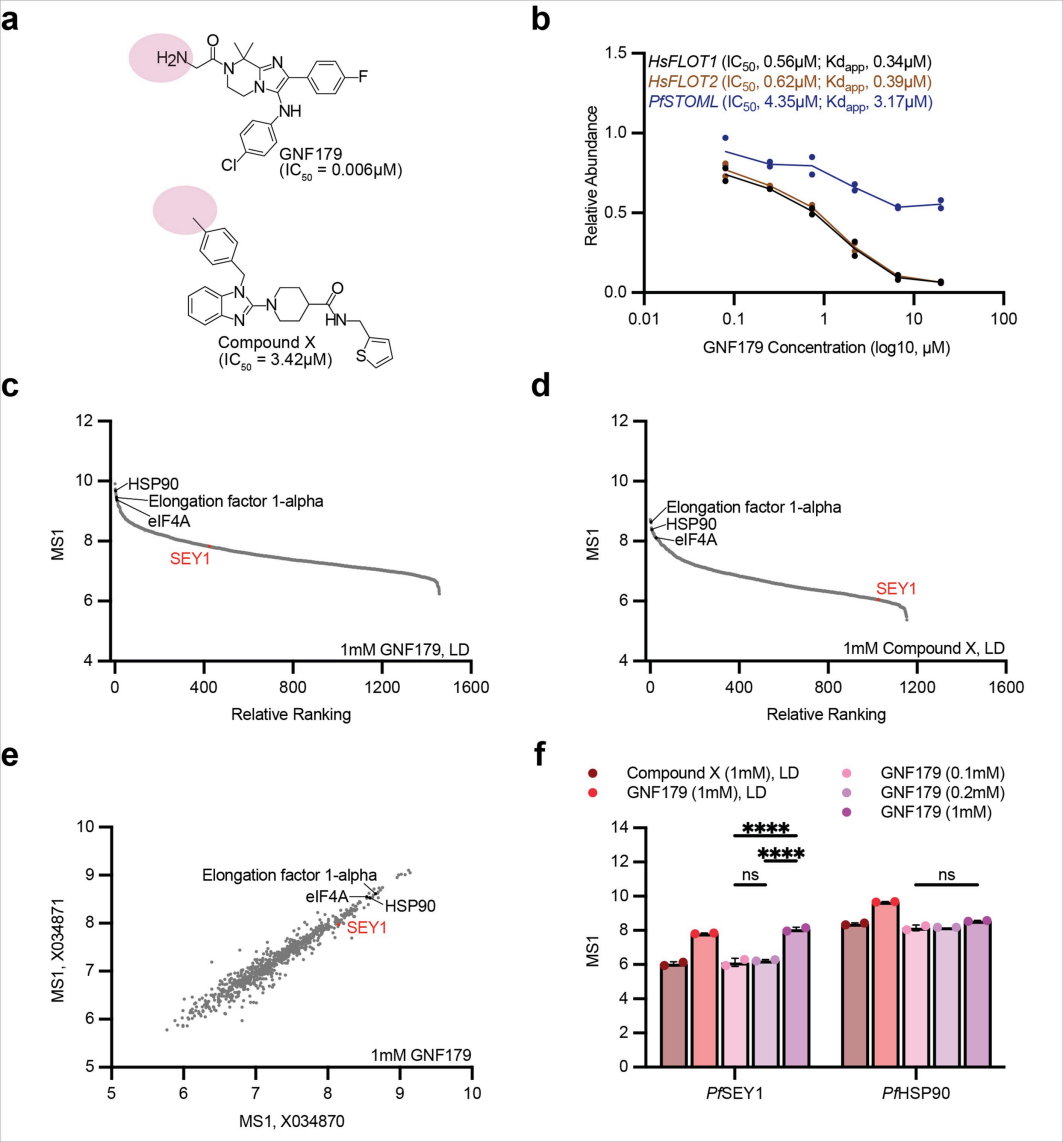
PfSEY1 is identified in proteomic affinity chromatography with GNF179-linked beads. **a.**. Compound structures of GNF179 and Compound X that were linked to beads for proteomic affinity chromatography; location of the linker is highlighted in pink. Their respective asexual blood stage IC_50_ measures are also displayed. **b.** Six-point dose response (with GNF179) to quantify GNF179 IC_50_ for *Hs*FLOT1, *Hs*FLOT2, and *Pf*STOML in experiments using beads coated with 0.2mM GNF179 (X035929 and X035930); line connects average relative abundance in each condition. IC_50_ and Kd_app_ averages of both experiments are also presented. **c.** Relative ranking of proteins by decreasing average MS1 scores for beads coated with 1mM GNF179 mixed with low detergent (LD) parasite protein extract (X035927, X035928); only the 1458 proteins detected in both experimental replicates are shown. **d.** Relative ranking of proteins by decreasing average MS1 scores for beads coated with 1mM Compound X mixed with low detergent (LD) parasite protein extract (X035202, X035203); only the 1154 proteins detected in both experimental replicates are shown. **e.** Distribution of MS1 scores for proteins detected with beads coated with 1mM GNF179 mixed with standard detergent protein extract (X034870, X034871); only the 774 proteins detected in both experimental replicates are shown. **f.** Distribution of MS1 scores for *Pf*SEY1 and *Pf*HSP90 across different bead conditions as described in Supplementary Table 2. On-bead concentrations for the compounds used in these tests are in parentheses.

To further investigate the MOA of IZPs and potentially identify a target, we first utilized proteomic affinity chromatography derived from the Cellzome platform (Bantscheff et al., 2007). Beads were linked to the amino group in GNF179 and to the 5-position of benzodiazol in MMV007564, a negative control compound that confers resistance to *PfCARL* mutations (Magistrado et al., 2016), generating Compound X. Both compounds maintained their inhibitory activity with IC_50_ measures of 0.006µM and 3.42µM, respectively (Fig. 1a). These compounds were tested in a series of 12 experiments, comprising biological duplicates of six different conditions. Low, membrane-preserving 0.02% NP40 protein extractions were mixed with 1mM GNF179 or Compound X, while standard 0.8% NP40 protein extractions were mixed with three different on-bead concentrations of GNF179 (1mM, 0.2mM and 0.1mM); mixed human cell (HEK293/K-562/Placenta/HepG2) extractions were also tested against 1mM GNF179 to rule out potential off-targets. For each experiment, tandem mass spectrometry (MS/MS) data were collected for 10 sample conditions (Supplementary Table 1).

Amongst the proteins detected (Supplementary Table 2), *Hs*FLOT1 and *Hs*FLOT2, ubiquitous human structural proteins involved in lipid raft and vesicle formation (Babuke et al., 2009), were most affected by GNF179. As a representative example, 0.2mM GNF179 on-bead conditions displayed IC_50_ averages of 0.56µM and 0.62µM for *Hs*FLOT1 and *Hs*FLOT2, respectively (Fig. 1b). We also measured apparent dissociation constants (Kd_app_) by considering protein depletion by the beads. Depletion factors are a function of the affinity of inhibitors to captured proteins and the concentration of tagged ligands. *Hs*FLOT1 and *Hs*FLOT2 were able to compete GNF179 off the beads, displaying Kd_app_ averages of 0.34µM and 0.39µM for *Hs*FLOT1 and *Hs*FLOT2, respectively (Fig. 1b). *Hs*FLOT1 and *Hs*FLOT2 *Plasmodium* orthologs, *Pf*RH2a and *Pf*STOML, respectively, were also identified, with the latter having generated IC_50_ and Kd_app_ averages of 4.35µM and 3.17µM, respectively (Fig. 1b). Both proteins lack clear small molecule binding pockets and are a predicted to be nonessential in *P. falciparum* (Zhang et al., 2018). Although *Hs*FLOT1 and *Hs*FLOT2 cannot be ruled out, they are unlikely candidates for an inhibitor with high parasite cell selectivity and low human cell toxicity.

Given that no compelling candidates were identified (*i.e.*, essential parasite proteins with low Kd_app_), we reanalyzed this data alongside those derived from bioinformatic analyses of genes mutated during *in-vitro* evolution of *S. cerevisiae* and *P. falciparum*, ranking targets based on evidence of interaction with known determinants of resistance, druggability, predictability of essentiality in *P. falciparum* (Supplementary Table 3), and localization. Many targets have evidence of ATP- and GTP-binding domains. Regarding the major *P. falciparum* resistance mechanisms of KAF156 and GNF179, *Pf*CARL was not identified in the proteomic pulldowns, but spectra for *Pf*ACT and *Pf*UGT were detected (Supplementary Table 2).

**Figure 2:**
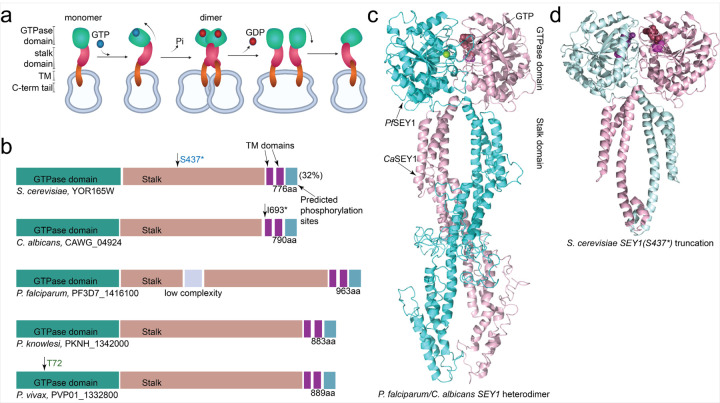
SEY1 expresses a structurally conserved GTPase protein amongst various species. **a.** Model recapitulating *S. cerevisiae* SEY1-mediated ER membrane fusion (Yan et al., 2015). The N-terminal GTPase domain is followed by the stalk domain, two transmembrane (TM) domains anchoring into ER membranes, and then the C-terminal tail. When guanosine triphosphate (GTP) is hydrolyzed, phosphate (Pi) is released, resulting in guanosine diphosphate (GDP). **b.** Domain structures for different SEY1 orthologs discussed in this paper. Arrows point to transmembrane (TM) domains, predicted phosphorylation sites within the C-terminal tail, truncation sites associated with GNF179 resistance (blue text), or MGLTools-predicted KAF156-interacting sites (green text); I693* refers to the nonsense mutation displayed in the *C. albicans* SEY1 crystal structure (PDB: 5CA9). **c.** Dimer structure between aforementioned truncated *C. albicans* SEY1 (*Ca*SEY1) and *P. falciparum* SEY1 (*Pf*SEY1) SWISS-derived homology model displays mirror symmetry. **d.** Predicted dimer structure of *S. cerevisiae* SEY1 that displays the S437* nonsense mutation conferring GNF179 resistance (LaMonte et al., 2020).

*Pf*SEY1 (PF3D7_1416100) was one of the highest ranked proteins, being druggable, pulled down abundantly, and parasite-specific; it is also predicted to be essential (Zhang et al., 2018). SEY1, or Synthetic Enhancer of YOP1, has been identified in *S. cerevisiae* as a dynamin-like GTPase, playing a role in homotypic ER membrane fusion (Fig. 2a): two *Sc*SEY1 proteins in different ER membranes bind GTP, initiating dimerization and subsequent membrane tethering upon GTP hydrolysis; GDP then releases, the proteins dissociate, and the process repeats (Yan et al., 2015). In *Fusarium graminearum* and *Candida albicans*, *SEY1* deletion reduces fungal virulence (Chong et al., 2020; Yan et al., 2015). Importantly, SEY1 is structurally conserved (Fig. 2b), with the high-quality crystal structure (minus transmembrane domains) of *C. albicans* SEY1 (Yan et al., 2015) displaying mirror symmetry with the *Pf*SEY1 model (Fig. 2c). There is also a druggable catalytic region, the GTPase domain, which comprises conserved motifs – the P-loop, Walker A, Walker B, and guanosine-binding sites (Hu et al., 2009).

In contrast to other *P. falciparum* proteins that appeared in almost all conditions (*e.g.*, HSP90, eIF4A, elongation factor 1-alpha), *Pf*SEY1 was specific to GNF179-linked beads, as more of it was pulled down by GNF179-linked beads (Fig. 1c) in the low detergent parasite extraction relative to Compound X-linked beads (Fig. 1d). This pulldown of *Pf*SEY1 by GNF179 is further corroborated with tests on the standard detergent parasite extraction (Fig. 1e). PfSEY1 also displayed a dose dependency with GNF179, as less protein was bound at lower on-bead concentrations; this contrasts with proteins that were uniformly detected in all GNF179 on-bead concentrations, such as *Pf*HSP90 (Fig. 1f). Moreover, SEY1 human orthologs within the atlastin family ranked lower (Supplementary Table 2), suggesting parasite specificity. Unfortunately, *Pf*SEY1 showed little evidence of being competed off GNF179-linked beads, with its relative abundance remaining consistent across all sample conditions (Supplementary Table 2).

### ScSEY1 mutations confer resistance to GNF179 in yeast

**Figure 3:**
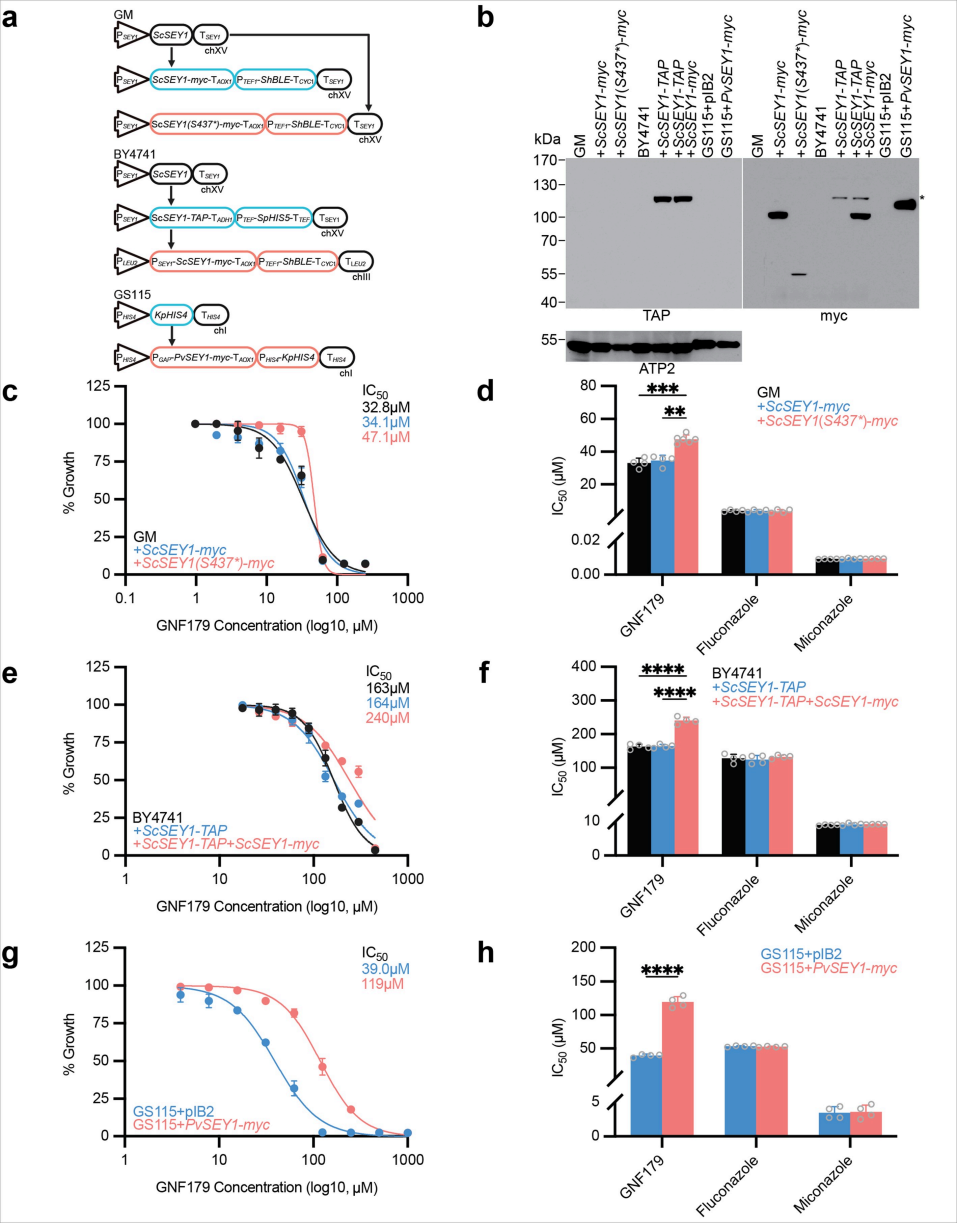
SEY1 mutations confer resistance to GNF179. **a.** Diagram of yeast mutants used in this study. *S. cerevisiae* Green Monster (GM) mutant was transformed to express either myc-tagged full-length *ScSEY1* or truncated ScSEY1(S437*) at the *ScSEY1* locus in chromosome XV (chXV). *S. cerevisiae* BY4741 mutant expressing TAP-tagged full-length *ScSEY1* at the *ScSEY1* locus is from the Yeast-TAP Tagged ORF Library (Ghaemmaghami et al., 2003); this mutant was transformed to express an additional myc-tagged *ScSEY1* at the *leu2* locus in chIII. *K. phaffii* GS115 was transformed to express myc-tagged full-length *PvSEY1* at the *his4* locus in chI. *BLE* and *HIS* genes are selection markers. **b.** Western blot of protein lysates from the yeast mutants used in this study. Left panel displays the membrane first probed with anti-TAP antibody. In the right panel, the same membrane was stripped and probed with anti-myc antibody; the asterisk marks residual TAP signal. For loading control, the membrane was stripped again and probed with anti-ScATP2 antibody that recognizes the 54kDa protein; this antibody also cross-reacts with *K. phaffii* ATP2. **c, d.** Dose response curve for GM+ScSEY1(S437*)-myc mutant treated with GNF179; average IC_50_ is presented. Bar graph summarizing IC_50_ averages for this mutant treated with GNF179 or antifungal controls. Data for GM and GM+*ScSEY1-myc* are also included. Results reflect at least two biological and technical replicates analyzed using Tukey test, noting comparisons with p-value<0.05. **e, f.** Dose response curve for BY4741 mutant expressing two copies of *ScSEY1* treated with GNF179; average IC_50_ is presented. Bar graph summarizing IC_50_ averages for this mutant treated with GNF179 or antifungals. Data for BY4741 and BY4741+*ScSEY1-TAP* are also included. Results reflect biological and technical duplicates analyzed using Tukey test, noting comparisons with p-value<0.05. **g, h.** Dose response curve for GS115*+PvSEY1-myc* mutant treated with GNF179; average IC_50_ is presented. Bar graph summarizing IC_50_ averages for this mutant treated with GNF179 or antifungals. Data for GS115 transformed with parental pIB2 plasmid are also included. Results reflect biological and technical duplicates analyzed using Sidak test, noting comparisons with p-value<0.05.

*SEY1* attracted our attention because previous *S. cerevisiae* selections with GNF179 identified a ScSEY1(S437*) nonsense mutation (Fig. 2d), albeit the mutation was found amongst additional mutations (LaMonte et al., 2020). To confirm the impact of this SNV, we utilized homologous recombination to replace native *ScSEY1* with myc-tagged full-length or truncated *ScSEY1* in the haploid Green Monster, or GM (Fig. 3a), that was used in the original selections. This attenuated strain has its 16 ATP-binding cassette (ABC) multidrug transporter genes replaced with modified *Aequorea victoria GFP* (Suzuki et al., 2011), thus requiring less compound for growth inhibition. Successful integration was confirmed by Western blot, displaying the expected 92kDa and 52kDa signals for GM + *ScSEY1-myc* and GM + ScSEY1(S437*)-myc, respectively (Fig. 3b, Supplementary Fig. 1). Dose-response testing in which wild-type GM and the GM + ScSEY1-myc mutant cells were grown with increasing GNF179 concentrations revealed similar IC_50_ measures of 32.8µM +/− 3.0µM and 34.1µM +/− 3.3µM, respectively, indicating that the fusion tag had no confounding effects. Contrarily, for the GM + ScSEY1(S437*)-myc mutant, we observed a 1.4-fold increase in IC_50_ to 47.1µM +/− 2.6µM (Fig. 3c). As a control, all strains were treated with fluconazole and miconazole, antifungals not expected to involve *SEY1*, and we observed similar IC_50_ measures between the strains (Fig. 3d).

### Additional SEY1 copies also confer resistance to GNF179

Copy number variations (CNVs) have also been associated with adaptation and drug resistance in parasites (Qidwai, 2020). Thus, we tested whether additional copies of *SEY1* affects GNF179 IC_50_ using the yeast model. To do so, we engineered BY4741, the parental strain of GM, that was already expressing the *ScSEY1-TAP* construct to also express an additional *ScSEY1-myc* construct (Fig. 3a); this mutant was subsequently confirmed via Western blot, displaying the expected 110kDa and 92kDa signals for TAP- and myc-tagged *Sc*SEY1, respectively (Fig. 3b). The mutant with two copies of the *ScSEY1* gene displayed a 1.5-fold increase in GNF179 IC_50_ to 240µM +/− 10µM, indicating a resistance phenotype (Fig. 3e). In contrast, wild-type BY4741 and the BY4741 + *ScSEY1-TAP* mutant treated with GNF179 displayed similar IC_50_ measurements of 163µM +/− 8µM and 164µM +/− 6µM, respectively, again indicating the absence of any fusion tag cassette effects. The IC_50_ measures are elevated here as the strains are expressing all ABC transporters. As expected, no strains showed increased resistance to the fluconazole and miconazole controls (Fig. 3f).

We also determined if the *Plasmodium SEY1* ortholog were sufficient to confer resistance against IZPs. Myc-tagged *P. vivax SEY1* under the glyceraldehyde-3-phosphate dehydrogenase promoter was transformed into wild-type *Komagataella phaffii* GS115, a yeast strain frequently used for recombinant protein expression (Fig. 3a); *PvSEY1* was used as it contains fewer low-complexity regions relative to *P. falciparum*. Integration was subsequently confirmed via Western blot, showing the expected 105kDa signal (Fig. 3b). Additionally, whole genome sequencing of these two lines confirmed the integration and showed no additional genomic changes outside of the added *PvSEY1-myc* construct (Supplementary Table 4). Compared to the parent, addition of *PvSEY1* yielded a 3.0-fold increase in GNF179 IC_50_ to 119µM +/− 8µM, from 38.9µM +/− 2.3µM (Fig. 3g). No differences in IC_50_ measures were observed between the strains when treating with fluconazole or miconazole (Fig. 3h). These results from two species of yeast and two orthologs of *SEY1* confirm that SEY1 mutations confer resistance to IZPs, but not to antifungals.

### GNF179 destabilizes PvSEY1 in protein lysates

**Figure 4:**
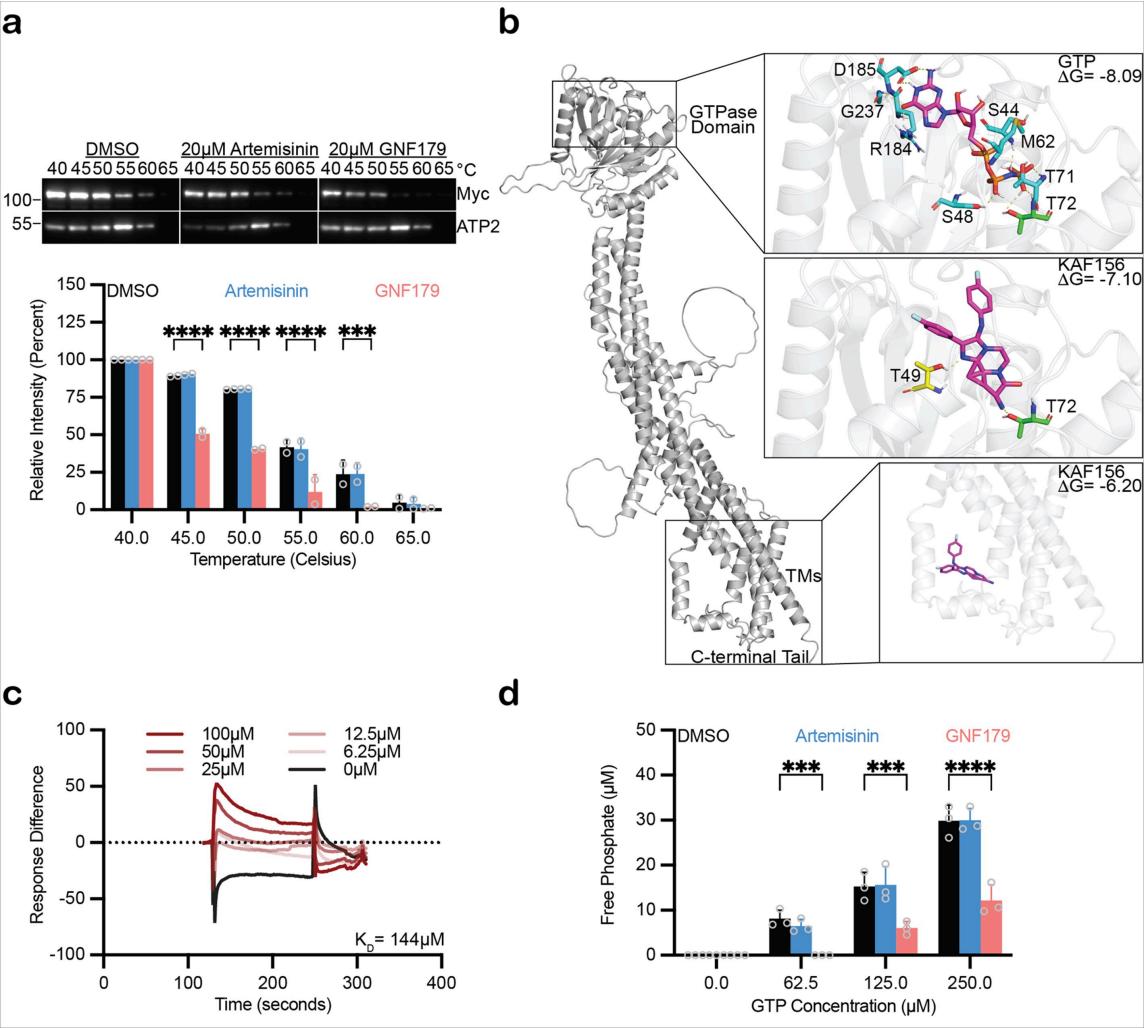
GNF179 interacts with Plasmodium SEY1, destabilizing the protein and blocking its GTPase activity. **a.** Representative Western blot of the abundance of *Pv*SEY1-myc remaining in whole cell lysates from GS115+*PvSEY1-myc* after treatment with 20µM artemisinin or GNF179, and subsequent thermal challenge from 40°C to 65°C. Data from biological duplicates are normalized against *Kp*ATP2 loading control protein and analyzed using Dunnett test, noting comparisons with p-value<0.05. **b.** Docking analysis of KAF156 and GTP onto the GTPase domain of the *Pv*SEY1 homology model derived from *Ca*SEY1 (PDB: 5CA9). GTP interacts with residues in cyan, whereas KAF156 interacts with residues in yellow; green residues interact with both ligands. Yellow dashes show interacting atoms, all within five angstroms. KAF156 was also docked onto the C-terminal tail of the *Pv*SEY1 homology model. **c.** Surface plasmon resonance analysis for the interaction between recombinant His-tagged *Pk*SEY1 and increasing GNF179 concentration; K_D_ is presented. **d.** GTPase activity of *Pv*SEY1-myc in protein lysates from *E. coli* is measured by quantifying free phosphate production in the presence of 125µM GNF179 or artemisinin. Data represent biological triplicates analyzed using Tukey test, noting comparisons with p-value<0.05.

Though targets of antimalarials are expected to be essential, the presence of a nonsense mutant, as opposed to a missense mutation, remained perplexing. We thus sought to further assess the GNF179-SEY1 interaction through cellular thermal shift assay (CETSA). This approach can be used against a specific candidate for target confirmation, relying on the principle that proteins bound to a compound may be protected from denaturation after temperature challenge (Dziekan et al., 2019). We utilized this method to determine if *Plasmodium* SEY1 interacts with GNF179. Protein lysate containing *Pv*SEY1-myc was extracted from GS115 and challenged to a range of temperatures in the presence of GNF179 or artemisinin control at a 100:1 compound-to-protein ratio. The subsequent samples were cleared of protein aggregates caused by thermal challenge and analyzed via Western blot, probing with anti-myc antibody. *Pv*SEY1-myc denaturation profiles for DMSO and artemisinin were similar after normalization against *Kp*ATP2, a loading control protein (Snyder et al., 1999) not expected to interact with GNF179. Unexpectedly, for GNF179, a severely denatured protein profile was observed: at 45°C, 89.2% and 90.4% of the protein remained in solution for DMSO and artemisinin, respectively, but only 50.7% of the protein for GNF179. This decline was also seen at 50°C (80.2%, 80.6%, and 40.6% for DMSO, artemisinin, and GNF179, respectively), 55°C (41.6%, 40.5%, and 11.8%), and 60°C (23.7%, 23.8%, and 2.0%) challenges (Fig. 4a, Supplementary Fig. 2). Although the premise of CETSA is that proteins are stabilized by compound binding, there are exceptions: human multidrug-resistance transporter 1 (*HsMDR1*) is destabilized by elacridar, which blocks ATP binding that normally stabilizes the transporter. Thus, in some situations, destabilization may be viewed as evidence of drug-target interaction (Reinhard et al., 2015). Perhaps GNF179 prevents SEY1 homodimerization, resulting in a lower melting temperature.

### KAF156 docks onto conserved PvSEY1 GTPase motifs

Since GNF179 destabilization of *Pv*SEY1 was observed, we hypothesize that GNF179 may block GTP binding that stabilizes *Pv*SEY1, akin to elacridar blocking of ATP binding that stabilizes *Hs*MDR1. We sought to address this by identifying potential binding sites through molecular docking, a technique that predicts binding between ligands and receptor residues. Without published crystal structures for *Pv*SEY1, we utilized ColabFold (Mirdita et al., 2022) to predict its structure, using truncated *Ca*SEY1 (RCSB: 5CA9) as a template. Using MGLTools (Eberhardt et al., 2021; Trott & Olson, 2010), KAF156 and GTP were fit into the GTPase domain of the monomeric *Pv*SEY1 homology model. Hydrogen bond interactions were predicted between GTP and *Pv*SEY1 Ser48, Thr72, Arg184, and Arg185 residues, conserved motifs of the GTPase domain (Yan et al., 2015). Hydrogen bond interactions were also predicted between KAF156 and the *Pv*SEY1 Thr72 residue that was observed with GTP. These results suggest that KAF156 docks onto the Walker A site of the GTPase domain. Additionally, the binding energies for GTP and KAF156 onto *Pv*SEY1 are − 8.09 and − 7.10 kcal/mol, respectively, further supporting the interaction (Fig. 4b).

### Recombinant PkSEY1 interacts weakly with GNF179

We further explore *Plasmodium* SEY1 interaction with IZPs through binding affinity experiments. Surface plasmon resonance (SPR) is a tool that elucidates physical protein-ligand interactions, with the benefit of measuring an equilibrium dissociation constant, K_D_, as observed for the inhibition of *Pf*HSP70 and *Pf*HOP by 2-phenylthynesulfonamide (Muthelo et al., 2022). Of note, we used recombinant *Plasmodium knowlesi* SEY1, as ganaplacide was even more potent against this species (van Schalkwyk et al., 2021). His-tagged *Pk*SEY1 was immobilized onto an NTA-coated sensor chip, and then GNF179 was flowed over at varying concentrations; plane-polarized light was then directed towards the metal surface of the chips and the resonant angle measured, as refractive indexes change with molecular interactions. As GNF179 concentration increased, there was a greater spike in response differences. A K_D_ of 144µM was measured for *Pk*SEY1-His (Fig. 4c), suggestive of weak binding affinity.

### GNF179 inhibits P v SEY1 GTPase activity in protein lysates

If IZPs were to bind to the GTPase domain, then there should be a decrease in GTPase activity. To test this, we developed a GTPase activity assay for *Plasmodium* SEY1 based on an existing assay measuring free phosphate production by *Sc*SEY1 (Anwar et al., 2012). Protein lysate containing *Pv*SEY1-myc-His was extracted from *Escherichia coli* and passed through a Ni-NTA column (Supplementary Fig. 3a). Each reaction comprising 1µM enzyme-containing eluate, 125µM artemisinin or GNF179, and 0µM to 250µM GTP was incubated for 30 minutes before measuring absorbance at 360nm wavelength. In the absence of GTP, no free phosphates were produced, as expected. Also, free phosphate levels between the DMSO and artemisinin conditions remained similar at all concentrations of GTP: 15.2µM +/− 3.19µM and 15.6µM +/− 4.1µM, respectively, at 125µM GTP, and 29.8µM +/− 3.5µM and 29.9µM +/− 2.7µM, respectively, at 250µM GTP. However, for the GNF179 condition, free production was hindered: 6.02µM +/− 1.49µM at 125µM GTP and 12.2µM +/− 3.5µM at 250µM GTP (Fig. 4d). As a control, *PvSEY1* with a Walker A (switch1) site mutation that inhibits GTPase activity (Yan et al., 2015) was tested. Mutated *Pv*SEY1(T7A) hydrolyzed significantly less GTP relative to *Pv*SEY1–3.79µM +/− 3.11µM vs. 14.8µM +/− 4.3µM with 250µM GTP and 5.63µM +/− 2.18µM vs. 27.1µM +/− 1.4µM with 500µM GTP – indicating enzymatically active *PvSEY1* in lysate (Supplementary Fig. 3b). This also supports the predicted IZP docking onto this residue (Fig. 4b). *E. coli* biotin ligase, which was the initial gene in the vector, was also tested for GTPase activity. Free phosphates were not produced by this control protein between 0µM and 250µM GTP (Supplementary Fig. 3c). These results indicate a reduction of *Pv*SEY1 GTPase activity by GNF179.

### GNF179 induces P. falciparum ER and Golgi morphology defects

**Figure 5:**
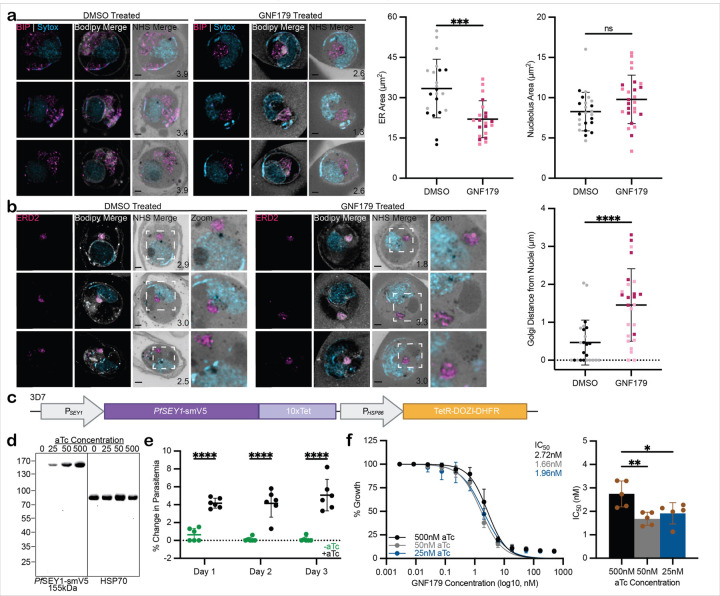
GNF179 affects Plasmodium ER and Golgi morphology. **a, b.** Ultrastructural expansion microscopy experiments tracking ER (*Pf*BIP), Golgi (*Pf*ERD2), and nucleus (Sytox) in the presence or absence of GNF179. ER area and nucleolus area (a) and Golgi distance from the nucleus (b) are measured for at least 20 cells from biological duplicates (represented by two-tone colors) and analyzed using unpaired t-test. Scale bar is 2µm; image depth is in microns indicated by values in the bottom right corner of NHS-merged images. **c.** TetR-DOZI aptamers construct is integrated into the *PfSEY1* locus for knockdown studies. *PfSEY1* is fused with the smV5-tag, followed by the 10xTet domain. **d.** Western blot of *Pf*SEY1-smV5 abundance in whole cell lysates of *P. falciparum* after treatment with increasing aTc concentrations. Right panel depicts the 155kDa constructed protein detected with anti-smV5 antibody; on the left panel, anti-*Pf*HSP70 antibody is used to detect the 70kDa loading control protein. **e.** Growth curve of the *PfSEY1* knockdown mutant. Cultures containing no aTc (-aTc) or 500nM aTc (+aTc) started at 1% parasitemia on Day 0 and change in their parasitemia were measured by subtracting previous-day parasitemia from current-day parasitemia. Data represent six biological replicates analyzed using Sidak test, noting comparisons with p-value<0.05. **f.** GNF179 IC_50_ measures for the *PfSEY1-smV5* mutant treated with 25nM, 50nM, or 500nM aTc. Dose response data (left) are summarized in the bar graph (right); five biological replicates were analyzed using Dunnet test, noting comparisons with p-value<0.05.

Previous studies have shown that mutations in *ScSEY1* cause defects in ER formation and, to an extent, Golgi morphology (Okamoto et al., 2012). If GNF179 were to affect SEY1, then there could be a resultant disturbance to ER morphology, and possibly to the associating Golgi. We examined the phenotype of GNF179-treated *P. falciparum* using ultrastructural expansion microscopy, a method established for isotopically increasing the size of cell structures while maintaining the integrity of the proteome (Gambarotto et al., 2019; Liffner et al., 2023). Asexual blood stage parasites were synchronized and treated two to four hours post-invasion with 25nM GNF179 for 16 hours, and then subsequently analyzed using this high-resolution method with antibodies to both the ER (*PfBIP*) and the Golgi (*PfERD2*). Several clear phenotypic changes were evident. In DMSO, the ER expanded to encompass the nucleus, whereas in GNF179, the ER signal around the nucleus was reduced: the mean ER area for DMSO and GNF179 were 33.4µm^2^ +/− 10.9µm^2^ and 22.0µm^2^ +/− 6.9µm^2^, respectively (Fig. 5a). This contrasts the ER expansion observed in previous GNF179-treated *P. falciparum* cells (LaMonte et al., 2020). Perhaps nucleus area also diminished upon GNF179 treatment, but this was not the case (Fig. 5a). Upon GNF179 treatment, the Golgi was also affected, appearing detached from the nucleus: the mean Golgi distance from the nucleus for DMSO and GNF179 were 0.466µm +/− 0.591µm and 1.46µm +/− 0.96µm, respectively (Fig. 5b). These observations were statistically significant, confirming a morphology defect in the *P. falciparum* ER and Golgi in response to GNF179 treatment.

### Conditional knockdown of PfSEY1 confirms that it is essential

Drug targets need to be essential, and *PfSEY1* has only been predicted to be an essential gene (Zhang et al., 2018). We sought to confirm its essentiality by performing conditional knockdown experiments with the tetracycline repressor protein-development of zygote inhibited-aptamer system (TetR-DOZI aptamers) to test parasite viability. In the absence of anhydrotetracycline (aTc), the TetR-DOZI chimeric protein binds to the ten aptamers on the transcripts, resulting in their conversion to inactive messenger ribonucleoprotein particles (mRNPs); in the presence of aTc, the chimeric protein preferentially binds to this molecule, thus allowing for translation to proceed (Rajaram et al., 2020). The TetR-DOZI aptamers tag was transfected into the 3’ UTR of *PfSEY1* using CRISPR/Cas9 (Fig. 5c). Western blot probing with V5 antibody for the included smV5 tag (Viswanathan et al., 2015) confirmed the 155kDa signal when aTc is present (Fig. 5d). Parasites were grown with (+ aTc) and without (-aTc) aTc, and their parasitemia was measured daily. After 24 hours, parasite growth was already diminished in the -aTc condition; in contrast, in the + aTc condition, in which *PfSEY1* is still being translated, growth appeared normal (Fig. 5e). This observation of decreasing viability is associated with diminishing *Pv*SEY1 levels (Fig. 5d). Thus, *PfSEY1* is an essential gene.

As a final step, we determined the IC_50_ against GNF179 for this *PfSEY1-smV5*^*Tet*^ knockdown mutant over a range of aTc concentrations. It was not possible to generate a reproducible dose response curve at low concentrations of aTc (*e.g.*, 5nM aTc), as growth was already substantially suppressed prior to the added suppression observed over a range GNF179 concentrations relative to the vehicle control; using 25nM or 50nM aTc mitigated this growth attenuation (Supplementary Fig. 4), while still reducing *Pf*SEY1 levels (Fig. 5d). Dose response experiments at 25nM and 50nM aTc generated an IC_50_ measure of 1.96nM +/− 0.45µM and 1.66nM +/− 0.28µM, respectively; compared to the 500nM condition (2.72µM +/− 0.55µM), this indicates a small but reproducible shift increase in sensitivity to GNF179 (Fig. 5f), consistent with SEY1 playing a role in the mechanism of action of GNF179.

## Discussion

Here we present evidence that Plasmodium *SEY1* is a critical antimalarial drug target and may play a role in the mechanism of action of IZPs.

Although data from *S. cerevisiae* guided our analysis, GNF179 is over 1000-fold less potent against this species, relative to *P. falciparum* (LaMonte et al., 2020). In yeast, *ScSEY1* is a nonessential gene (Hu et al., 2009), as mutants devoid of *ScSEY1* do not show a growth defect nor defects in ER morphology; only when additional genes – *ScYOP1, ScRTN1* – are missing will aberrant ER morphology occur (Anwar et al., 2012). Furthermore, there are redundant pathways for ER membrane fusion: retrograde SNAREs can fuse ER membranes in the absence of *Sc*SEY1 (Rogers et al., 2013), or possibly when *Sc*SEY1 is sequestered by IZPs. This could explain the higher GNF179 IC_50_ measures with yeast (Fig. 3c-h). The fact that a nonsense mutation that removes *Sc*SEY1 transmembrane domains confers resistance to IZPs suggests that presence of a GNF179/*Sc*SEY1 complex is more toxic than having no *Sc*SEY1 at all.

We had previously shown that parasites are more sensitive to GNF179 when protein export is altered. Additionally, GNF179 misfolding of proteins in the ER could trigger the ERAD(II) pathway of degradation, which depends on autophagy and lysosomal trafficking (LaMonte et al., 2020). In Dictyostelium *discoideum*, amoeba mutants devoid of *SEY1* demonstrate a constitutive activation of the unfolded protein response pathway (Husler et al., 2021). Furthermore, mutating *SEY1 Arabidopsis thaliana* ortholog Root Hair Defective 3 (*AtRHD3*) changes Golgi distribution and motility within the plant (Chen et al., 2011). *Pf*SEY1 has many characteristics of a predicted target of IZPs, and we provide additional data implicating protein trafficking in IZP function.

Since GNF179 resistance was observed with the ScSEY1(S437*) nonsense mutant, we cannot ignore possible direct binding sites of IZPs to the C-terminal tail of the protein. However, docking KAF156 onto the C-terminal region of *Pv*SEY1 did not yield any interactions (Fig. 4b). The C-terminal tail may still be important to IZP binding. Mutating the phosphorylated residues within the C-terminal tail of *At*RHD3 aberrates tubule formation (Ueda et al., 2016). SEY1 human ortholog atlastin-1 (*Hs*ATL-1) possesses an amphipathic helical domain at the C-terminus that assists in facilitating membrane fusion (Faust et al., 2015). IZPs could also misfold SEY1 in a manner preventing its C-terminal TM domains from embedding into specific lipid membranes, which is required in *Sc*SEY1-dependent ER membrane fusion (Sugiura & Mima, 2016).

Although our data indicate a genetic interaction between *SEY1* and GNF179, as well as protein-ligand binding, the measured K_D_ (Fig. 4c) may not be sufficient to account for the low nanomolar inhibitory activity of IZPs, unless there is potentiation of activity in the cellular environment that cannot be reproduced *in-vitro*. For example, the ER membrane or specific lipids could be required to facilitate binding (Sugiura & Mima, 2016; Wang & Rapoport, 2019). There could also be additional regulation, such as C-terminal tail phosphorylation, that is needed to fully capture binding. To further elucidate this, experiments involving *Plasmodium* SEY1-GNF179 protein crystallography and *in-vivo* tracking must be conducted.

Like other drug targets, *PfSEY1* is predicted to be an essential gene in *P. falciparum* (Zhang et al., 2018). This was confirmed with our *PfSEY1-smV5*^*Tet*^ knockdown experiments, in which cell growth ceased within 24 hours of aTc-dependent translation inhibition (Fig. 5e). Like other drug targets, it contains a known druggable pocket, the nucleoside triphosphate hydrolase domain. Previous studies have shown kinases and tRNA synthetases possessing this domain to be inhibited by antimalarials (Arendse et al., 2021; Xie et al., 2023). IZPs could still target a yet to be discovered GTPase involved in protein trafficking. Even if *PfSEY1* were not the target of IZPs, it is still a compelling target for future drug development.

IZPs remain the leading antimalarial candidate, being farthest along the drug development pipeline with a novel mode of action. Currently, no SNPs conferring IZP resistance have been found in African samples (Foguim et al., 2019). Moreover, previous studies have shown that they inhibit many species of *Plasmodium* (Aniweh et al., 2023; Dembele et al., 2021; van Schalkwyk et al., 2021). Ultimately, investigating IZP effects on different SEY1 orthologs strengthens this new compound-protein interaction. Through target identification, resistance to IZPs can be better anticipated, toxicity can be further remedied, and synergistic drug research can be more guided.

## Materials and Methods

### Proteomic affinity chromatography

Setup is summarized in Supplementary Table 1. GNF179- and Compound X-coated beads were mixed with *P. falciparum* protein lysates (extracted using low 0.02% or standard 0.8% detergent) and subsequently competed with free GNF179; mixed human cell extracts were also tested. Ten samples were analyzed in parallel (TMT 10-plex) to generate values for the affinity of the beads to the bound proteins (“depletion” values, four samples) and to generate IC_50_ values (six samples) in a single experiment. Sample 1 and 2 represent the vehicle control; sample 3 and 4 were done in the same way, but while the beads were discarded after the first incubation step the extract was incubated with fresh beads to measure how much protein could rebind to the fresh beads (was depleted from the extract by first bead-binding). Apparent dissociation constants were determined by considering the protein depletion by the beads. Sample 5–10 were used to generate IC_50_ values by adding GNF179 over a range of concentrations (maximum of 20µM, 1:3 dilutions). MS/MS was utilized for quantification. MS1 score analysis was done using Prism 10.

### Strain construction

For *S. cerevisiae* transformation, 8 OD_600_ early-log phase cells (0.8 OD_600_ mL^−1^) were washed twice with cell-grade water and then twice with lithium acetate buffer (100mM lithium acetate, 10mM Tris-HCl pH 7.5, 1mM EDTA). Approximately 100µg boiled salmon sperm DNA and 500ng PCR-generated linear DNA were added, followed by six-fold volume of 40% v/v polyethylene glycol in lithium acetate buffer. The sample was incubated at 30°C for 30 minutes, treated with 10% v/v DMSO, and subsequently heat-shocked at 42°C for 15 minutes. Cells were sedimented and resuspended in 1mL YPD (1% yeast extract, 2% peptone, 2% dextrose), allowing overnight recovery at 30°C before plating on YPD+Zeocin (50µg mL^−1^) agar plates. SDS-PAGE and subsequent Western blot with mouse anti-myc (Invitrogen, R950–25, 1:1000) or rabbit anti-TAP (Invitrogen, CAB1001, 1:1000) antibodies detected the proteins of interest; rabbit anti-ScATP2 (gifted by the Subramani Lab at UCSD, 1:2000) was included as a loading control. HRP-linked goat anti-mouse (Bio-Rad, 1706516, 1:5000) and goat anti-rabbit (Bio-Rad, 1706515, 1:5000) antibodies were the secondary antibodies used for chemiluminescence. The mutant strains used in this study can be found in Supplementary Table 5.

The three linear DNA constructs transformed into *S. cerevisiae* were created using oligos summarized in Supplementary Table 6. Myc-tagged full-length *ScSEY1* was generated using oligos oKC1 and oKC2 on pPICZ-C plasmid (gifted by the Subramani Lab). Myc-tagged truncated ScSEY1(S437*) was generated using oligos oKC3 and oKC2 on the same plasmid. Oligos oKC4 and oKC5 were used on genomic DNA (gDNA) from the *ScSEY1-myc* mutant to obtain the additional *ScSEY1* gene construct that was transformed into BY4741+*ScSEY1-TAP*, which is from the Yeast-TAP Tagged ORF Library (Ghaemmaghami et al., 2003).

For *K. phaffii* (gifted by the Subramani Lab) transformation to express *P. vivax SEY1*, 50 OD_600_ early-log phase cells were resuspended in 5mL YPD containing 20mM HEPES pH 8.0 and 25mM 1,4-Dithiothreitol and then incubated at 30°C for 15 minutes (with orbital rotation). After, cells were washed three times with cold sterile, and then with cold 1M sorbitol; the cells were incubated in 500µL cold 1M sorbitol for one hour. Exactly 100µL of cells were mixed with 500ng linearized plasmid DNA. After 10 minutes on ice, the sample was placed in a 2mm cuvette and electroporated (50μF, 200Ω, 7500kV cm^−1^). Cells were plated on SD+CSM-His (0.17% yeast nitrogen base, 0.5% ammonium sulfate, 2% dextrose, 0.077% CSM-His) agar plates. Strains underwent quality assurance using SDS-PAGE and subsequent Western blot with mouse anti-myc and HRP-linked goat anti-mouse antibodies.

The plasmid DNA transformed into *K. phaffii* was generated by amplifying myc-tagged codon-optimized *PvSEY1*, which was ordered from IDT, with oligos oKC6 and oKC7. The pIB2 plasmid (gifted by the Subramani Lab) was digested with EcoRI and KpnI restriction enzymes. Gibson assembly (NEB, E2611) was used to combine these two gene fragments. The complete plasmid was linearized with StuI restriction enzyme.

For transformation of Rosetta 2 DE3 (Novagen, 71400) and DH5-alpha strains of *E. coli* (NEB, C2987), 50μL of chemically competent cells were mixed with 10ng circular plasmid or 10% of a Gibson reaction. Cells were incubated on ice for 30 minutes before heat-shocking at 42°C for 30 seconds. After incubation on ice for five minutes, cells were added to 950μL SOC and incubated at 37°C for an hour (with shaking at 225rpm). Cells were plated on LB+Ampicillin (0.5% yeast extract, 1% tryptone, 1% NaCl, 100µg mL^−1^ ampicillin) plates. Plasmids were extracted using miniprep kits (Promega, A1460) and sequenced by Plasmidsaurus for accuracy.

For construction of the *PfSEY1-smV5*^*Tet*^ line, 25μg homology-directed repair (HDR) plasmid was linearized by digestion, purified, and then co-transfected with 20μg guide RNA (gRNA) plasmid (containing *Streptococcus pyogenes Cas9*) into *P. falciparum* 3D7 parasites (synchronized as schizonts) using the Amaxa 4D system. Parasites were cultured with 500nM anhydrotetracycline (aTc) from the onset of transfection. One day post transfection, 2.5nM WR99210 (Jacobus Pharmaceuticals) drug pressure was applied.

*HDR (pAM115) and gRNA plasmids (pAM116, pAM117, pAM118):* 3’- and 5’-homology regions (HR) of *PfSEY1* were amplified from *P. falciparum* 3D7 gDNA using oligos oJDD6152/6153 and oJDD6146/6147, respectively. A codon-altered region for the last 88 amino acids was generated by IDT and amplified using oligos oJDD6148/6149. The smV5^Tet^-Dozi-DHFR drug cassette region and pGEM backbone were amplified from pPG03 (Gurung et al., 2024) using oligos oJDD6150/6151 and oJDD6154/6155, respectively. Fragments were joined using the NEBridge Golden Gate method (with NEB BsaI-HF v2) by New England Biolabs. To generate the three *PfSEY1* gRNA plasmids, oligos corresponding to each guide were annealed and ligated into BpiI-digested pRR216 (Rudlaff et al., 2019), which encodes *Sp*Cas9 and a U6 guide cassette. For pAM116, oligos oJDD6156/6157 were used. For pAM117, oligos oJDD6158/6159 were used. For pAM118, oligos oJDD6160/6161 were used (Supplementary Table 6).

### Protein extraction

Protein lysate containing functional *Sc*SEY1-myc was extracted from *S. cerevisiae*, while protein lysate containing functional *Pv*SEY1-myc was extracted from *K. phaffii*. Approximately 400 OD_600_ early-log phase cells were washed twice with sterile water and incubated in 8mL zymolyase buffer (500mM KCI, 5mM MOPS-KOH pH 7.2, 10mM Na_2_SO_3_, 5000U Zymolyase-100T) for 30 minutes at 30°C (orbital rotation). Cells were then pelleted and resuspended in 4mL homogenization buffer (5mM MES, 1M sorbitol, 5mM NaF, 20mM EDTA, 1mM PMSF, 1x protease inhibitor cocktail) and lysed using a Dounce homogenizer. Cell debris was removed by centrifuging at 1,000g for 10 minutes. *Sc*SEY1 was solubilized in 0.8% digitonin, while *Pv*SEY1 protein was solubilized in 1% Triton X-100, for two hours at 4°C (with orbital rotation). Solubilized proteins were clarified by centrifugation at 100,000g for 20 minutes. Proteins were detected via Western blot using mouse anti-myc, rabbit anti-TAP, and rabbit anti-*Sc*ATP2 antibodies, along with the corresponding HRP-linked secondary antibodies.

Full-length functional *Pv*SEY1-myc-His protein was extracted from *E. coli.* Approximately 1L of early-log phase cells were harvested after induction with 1mM IPTG at 16°C for 16 hours (with shaking at 160rpm). Cells were lysed in 20mL lysis buffer (1% lysozyme, 1% protease inhibitor cocktail, 1% Triton X-100, 1mM PMSF, 20mM imidazole, 2500U endonuclease in B-PER reagent) at 4°C for two hours (with orbital rotation). The cell lysate, cleared by chilled centrifugation at 20,000g for 20 minutes, was incubated with Ni-NTA beads at 4°C for 16 hours (with orbital rotation) before passing them through a column. The beads were washed three times with wash buffer (500mM NaCl, 20mM Tris-HCl pH 7.9, 20mM imidazole). Then, proteins were eluted off the beads using two column volumes of elution buffer (500mM NaCl, 20mM Tris-HCl pH 7.9, 300mM imidazole). Protein samples were concentrated using Pierce protein concentrators (Thermo Scientific, 88502) and quantified via Qubit (Invitrogen, Q33211). Proteins were detected via Western blot, using mouse anti-myc and HRP-linked goat anti-mouse antibodies.

For quantifying *Pf*SEY1-smV5 abundance in knockdown experiments, whole cell lysates were extracted from *P. falciparum* cells that were harvested from red blood cells via saponin lysis. The parasites were boiled in Laemmli buffer containing beta-mercaptoethanol for five minutes; cell debris was removed via centrifugation at max speed for five minutes. Protein concentration was measured using Qubit before SDS-PAGE and Western blot with mouse anti-V5 (Invitrogen, R960, 1:1000), mouse anti-*Pf*HSP70 (Genscript, SC1320A 1:1000), and goat anti-mouse antibodies.

### IC_50_ determination

For proteomic affinity chromatography experiments, inhibition of intraerythrocytic *P. falciparum* growth was determined by a modified *in-vitro* [3H]-hypoxanthine incorporation method (Desjardins et al., 1979). Briefly, a culture of parasitized red blood cells (0.5% parasitemia with over 70% of total parasites in ring stage, 2.0% hematocrit) in RPMI-1640, 5% AlbuMAX, and 5μM hypoxanthine was exposed to drug serial dilutions. 96-well plates (Costar, #3894) were incubated for 24 hours at 37°C, 5% CO_2_, 5% O_2_, and 95% N_2_. After, [3H]-hypoxanthine was added, and plates were incubated for an additional 24 hours. Then, plates were harvested on glass fiber filters (Wallac, #1450–421) using a cell harvester (Tomtec 96, PerkinElmer). Filters were dried, and MeltiLex A melt-on scintillator sheets (PerkinElmer #1450–441) were used to determine [3H]-hypoxanthine incorporation. Radioactivity was measured using a MicroBeta counter (PerkinElmer). Data were normalized using the incorporation by the positive control (infected red blood cells without drug). IC_50_ values were determined using Excel and Grafit 5. Human biological samples (placenta in pull-downs, red blood cells in IC_50_ determination) were sourced ethically, and their use in the research was in accordance with terms from an IRB/REC-approved protocol.

For yeast experiments, a single colony was grown in YPD overnight at 30°C to early-log phase. Exactly 0.0005 OD_600_ of cells were grown in 100µL YPD and exposed to a ten-point dose response of GNF179 or antifungals. After incubation at 30°C for 16 hours (with shaking at 500rpm), OD_600_ was measured on a plate reader (BioTek Synergy). Percent growth was normalized against the vehicle-only condition (1% DMSO) and plotted against log_10_ compound concentration using Prism 10, generating curves and IC_50_ measures. Experiments were conducted in 96-well format, with at least two biological and technical replicates. Background strains tested include GM, a mutant *S. cerevisiae* strain devoid of 16 ABC multidrug transporter (Suzuki et al., 2011), *S. cerevisiae* BY4741, and *K. phaffii* GS115 (Supplementary Table 5).

For experiments with *P. falciparum PfSEY1-smV5*^*Tet*^, parasites were cultured in human O^+^ red blood cells at 2% hematocrit (BioIVT, Hicksville, NY) in prewarmed parasite growth media (RPMI-1640 with L-glutamine and 25mM HEPES, supplemented with 2g L^−1^ NaHCO_3_, 2.6g L^−1^ AlbuMAX II, 13.6mg L^−1^ hypoxanthine, and 50mg L^−1^ gentamicin). Selection of the mutant was maintained with 2.5nM WR99210, and normal wild-type levels of *Pf*SEY1 were maintained with 500nM aTc. Parasitemia was assessed by examining thin blood smears fixed with methanol and subsequently stained with Giemsa under an upright microscope (Olympus CX33). Asynchronous parasites in the presence of aTc and WR99210 were treated with a 12-point dose response of GNF179 (0.003–500nM) in 384-well V-bottom plate format. After 72 hours, growth media was removed and 2µM Syto-61 (Invitrogen, S11343) was added. Plates were read using a flow cytometer (BD FACSCanto) 20 minutes later. Data was visualized using FlowJo (BD Biosciences) and analyzed with Prism 10, generating curves and IC_50_ values. Experiments were conducted in biological and technical replicates and normalized against the DMSO-only condition.

### Whole genome sequencing

Yeast cells were grown overnight to early-log phase and gDNA was extracted using the DNeasy Blood and Tissue kit, following manufacturer’s protocol (Qiagen, 69504). DNA was diluted to 300ng mL^−1^ for DNA library preparation with Nextera XT (Illumina, FC-131–1024), barcoding with DNA/RNA UD Indexes Set A (Illumina, 20027213), and purification with AMPure XP beads (Beckman, A63880). Resulting purified tagmented DNA was pooled and submitted to the IGM Genomics Center at UCSD for Illumina MiSeq sequencing. Paired-end raw sequence reads were aligned to *K. phaffi* GCF_000027005.1 reference genome using BWA-MEM (Li & Durbin, 2009) and preprocessed following standard GATK version 3.5 protocols (McKenna et al., 2010; Van der Auwera et al., 2013). SNVs and indels were called with GATK HaplotypeCaller and filtered based on the following exclusion criteria: quality score (QUAL) <500, filtered depth (DP) <7.

### Thermal shift assay

Solubilized protein lysate from 400 OD_600_ early-log phase yeast cells was divided into three microcentrifuge tubes – one containing 20mM GNF179, another containing 20mM artemisinin, and a third containing DMSO. Each tube was then further divided into six PCR tubes, one for each temperature condition (40°C-65°C). Each tube was heated for two minutes, and then chilled for two minutes. Centrifugation at 100,000g for 20 minutes in 4°C removed denatured protein aggregates. Analysis of the supernatants was done by SDS-PAGE and subsequent Western blot with mouse anti-myc and rabbit anti-ScATP2 antibodies. Intensities were quantified using ImageJ and analysis was done using Prism 10.

### Molecular docking

To generate the theoretical *Pv*SEY1 structure, protein sequences were processed in ColabFold v1.5.1 (Mirdita et al., 2022), using *Ca*SEY1 (RCSB: 5CA9) as a template. The highest scoring model was then analyzed using MGLTools (Eberhardt et al., 2021; Trott & Olson, 2010). The protein structure was stripped of water molecules, then given Kollman charges and polar hydrogen atoms. Molecular structure for GTP or KAF156 was then uploaded. Autogrid 4 was executed around the GTPase domain or the C-terminal tail of predicted *Pv*SEY1 structure. Autodock 4 was then used to fit the ligand into these regions, generating a score for each position. PyMol was used to create protein models, noting hydrogen bonds between ligand and amino acid residues within five angstroms.

### Surface plasmon resonance

Full-length *P. knowlesi* SEY1 (His-tagged) purchased from MyBioSource was processed on a Biacore 3000 by Profacgen. *P*kSEY1-His ligand was immobilized on NTA sensor chips (washed with 350mM EDTA and 500mM NiSO_4_ prior) and washed with 0.05% surfactant P20 in 1x PBS to reach 1300 resonance units (RU). Various concentrations of the GNF179 analyte were then injected using the following parameters: 120 second contact time, 0.5 second dissociation contact time, 30µL min^−1^ flow rate. To obtain the K_D_, data were analyzed using BIAevaluation software.

### GTPase assay

GTPase activity was measured by quantifying free phosphate production using the EnzChek Phosphate assay kit (Invitrogen, E6646). The 100µL reactions comprise 50µL reaction buffer (20% glycerol, 2mM EDTA, 4mM β-mercaptoethanol, 5mM MgCl_2_, 50mM HEPES, 200mM KCl), 20µL MESG, and 1µL PNP; the remaining 29µL is composed of the following: GTP, compound or vehicle, 1µM enzyme. A_360_ measurements were taken after 30 minutes on a plate reader (BioTek Synergy), kept at 37°C. Free phosphate generated was plotted against increasing GTP or compound concentration. Assays were done in 96-well format, with biological and technical replicates. Analysis was done using Prism 10.

### Ultrastructural expansion microscopy

*P. falciparum* 3D7 parasites expressing Cas9 were tightly synchronized using a combination of density separation by 60% Percoll gradient and subsequent selective osmotic lysis after two hours of incubation to select for newly invaded parasites. Cultures were then treated with 25nM DMSO or GNF179 for 16 hours before samples at approximately 1% hematocrit were settled on poly-d-lysine-coated 12mm round coverslips for 30 minutes at 37°C, and then fixed by 4% PFA in PBS for 20 minutes at 37°C. Coverslips were washed three times with PBS before treating with 500µL of 1.4% v/v formaldehyde and 2% v/v acrylamide in PBS. Samples were incubated overnight at 37°C. Gelation, denaturation, staining, and expansion of the gels were performed as previously described (Liffner & Absalon, 2021). Gels were stained with rabbit anti-*Pf*BIP (gifted by the Dvorin Lab at Harvard Medical School, 1:2000) for ER or rabbit anti-*Pf*ERD2 (gifted by the Muralidharan Lab at the University of Georgia, 1:2000) for Golgi. Secondary antibody staining with goat anti-rabbit Alexa Fluor 488 (Thermo Scientific, A-11034, 8µg mL^−1^), NHS Ester Alexa Fluor 405 (Thermo Scientific, A30000, 8 µg mL^−1^), and SYTOX Deep Red (Thermo Scientific, S11380, 1µM) were used to stain proteins and DNA. Stained gels were imaged using a Zeiss LSM900 AxioObserver with an Airyscan 2 detector and 63x Plan-Apochromat objective lens with numerical aperture of 1.4. Images were analyzed using Zen Blue 3.5 software (Zeiss, Oberkochen, Germany) using 3D Airyscan processing at moderate filter strength. Images shown are maximum intensity projections of between 10 and 30 z-slices of the entire image. Nucleolus and ER area was calculated by taking the area of the projection that is showing the largest surface area of the respective signal. Distance of Golgi to nucleus was calculated by measuring the 3D distance of the closest point of the Golgi signal to that of the nuclear signal. Analysis was done on Prism 10.

### Parasite growth assay

A culture of *P. falciparum PfSEY1-smV5*^*Tet*^ grown under 2.5nM WR99210 selection pressure and 500nM aTc was washed with prewarmed parasite growth media three times to remove aTc. Quantitative growth assays were performed in 96-well V-bottom plates using asynchronous parasites set up in triplicate and cultured with 5, 25, 50 or 500nM aTc. All cultures, which started at 1% parasitemia, were checked daily by blood smears.

## Supplementary Material

Supplement 1
