# Supplementary material for: Plasmodium SEY1 is a novel druggable target that contributes to imidazolopiperazine mechanism of action
Source: Res Sq. 2024 Sep 23:rs.3.rs-4892449. Preprint. [Version 1] doi: 10.21203/rs.3.rs-4892449/v1 (PMC11469372; doi:10.21203/rs.3.rs-4892449/v1)
Supplement: Supplement 1 [file NIHPPRS4892449V1-supplement-1.pdf]

Supplementary Files

This is a list of supplementary files associated with this preprint. Click to download.

- SupplementaryFigureandtable135.docx
- IZPSuppTable2CellzomeALT.xlsx
- IZPSuppTable4.xlsx
- IZPSuppTable6.xlsx
